# Are Glucosylceramide-Related Sphingolipids Involved in the Increased Risk for Cancer in Gaucher Disease Patients? Review and Hypotheses

**DOI:** 10.3390/cancers12020475

**Published:** 2020-02-18

**Authors:** Patricia Dubot, Leonardo Astudillo, Nicole Therville, Frédérique Sabourdy, Jérôme Stirnemann, Thierry Levade, Nathalie Andrieu-Abadie

**Affiliations:** 1 INSERM UMR1037, CRCT (Cancer Research Center of Toulouse), and Université Paul Sabatier, 31037 Toulouse, France; patricia.dubot@inserm.fr (P.D.); rmhp.redaction@gmail.com (L.A.); nicole.therville@inserm.fr (N.T.); sabourdy.f@chu-toulouse.fr (F.S.); 2 Laboratoire de Biochimie Métabolique, Centre de Référence en Maladies Héréditaires du Métabolisme, Institut Fédératif de Biologie, CHU de Toulouse, 31059 Toulouse, France; 3 Service de Médecine Interne, CHU de Toulouse, 31059 Toulouse, France; 4 Service de Médecine Interne Générale, Hôpitaux Universitaires de Genève, CH-1211 Geneva, Switzerland; jerome.stirnemann@hcuge.ch

**Keywords:** glucocerebrosidase, sphingosine, ceramide, myeloma, melanoma, acid ceramidase, glucosylsphingosine

## Abstract

The roles of ceramide and its catabolites, i.e., sphingosine and sphingosine 1-phosphate, in the development of malignancies and the response to anticancer regimens have been extensively described. Moreover, an abundant literature points to the effects of glucosylceramide synthase, the mammalian enzyme that converts ceramide to β-glucosylceramide, in protecting tumor cells from chemotherapy. Much less is known about the contribution of β-glucosylceramide and its breakdown products in cancer progression. In this chapter, we first review published and personal clinical observations that report on the increased risk of developing cancers in patients affected with Gaucher disease, an inborn disorder characterized by defective lysosomal degradation of β-glucosylceramide. The previously described mechanistic links between lysosomal β-glucosylceramidase, β-glucosylceramide and/or β-glucosylphingosine, and various hallmarks of cancer are reviewed. We further show that melanoma tumor growth is facilitated in a Gaucher disease mouse model. Finally, the potential roles of the β-glucosylceramidase protein and its lipidic substrates and/or downstream products are discussed.

## 1. Introduction: Glucosylceramide and Gaucher Disease

In the last three decades, much attention has been paid to the functions of sphingolipids in cancer cell biology, with a major focus on the roles played by ceramide and sphingosine 1-phosphate (such an interest in this field is exemplified by the present Special Issues) [1,2,3]. Besides these simple sphingolipids, glycolipids are also involved in multiple facets of cancer development. For instance, as components of membrane microdomains, gangliosides, such as GM3, GD3 or GD2, have been extensively described as regulators of tyrosine kinase receptors with subsequent effects on tumor cell proliferation, migration and survival (for recent reviews, see [4,5]). More recently, the implication of several complex glycosphingolipids in the epithelial-to-mesenchymal transition process has also been documented (see, for instance, [6,7,8]). Of note, in addition to their roles in oncogenic signaling, some complex glycolipids can be used as prognostic markers in cancer disease progression. Furthermore, clinical trials with some monoclonal antibodies against specific tumor-associated complex glycolipids (e.g., anti-GD2) have shown improved outcomes in patients with solid cancers such as neuroblastoma [9].

In mammals, the vast majority of glycolipids are formed from β-glucosylceramide (GlcCer). How this lipid modulates cancer development still remains to be fully elucidated. In the late nineties, a pioneering work by Cabot and co-workers revealed the importance of the enzyme GlcCer synthase in promoting the resistance of breast cancer cells to various anticancer agents [10,11]. Since then, numerous studies have explored the role of ceramide glucosylation in multidrug resistance (for reviews, see [12,13]). However, how GlcCer catabolism may influence the behavior of cancer cells or tumor cell microenvironment has received little attention. Here, we review the knowledge in this field and discuss various hypotheses.

**Figure 1:**
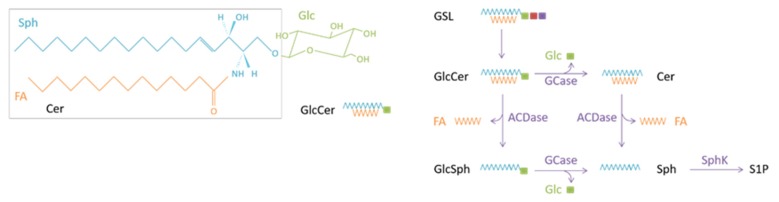
Glucosylceramide structure and metabolism. Abbreviations: Cer, ceramide; FA, fatty acid; Glc, glucose; GSL, glycosphingolipid; Sph, sphingosine; S1P, sphingosine 1-phosphate; SphK, sphingosine kinase. Fatty acids found in GlcCer usually include C16:0, C18:0, C22:0 and C24:1.

GlcCer is synthesized from ceramide by a single enzyme, GlcCer synthase, encoded in humans by the *UGCG* gene. At remarkable variance with other sphingolipid synthesis enzymes, GlcCer synthase faces the cytosolic surface of the Golgi apparatus. Using UDP-glucose as a sugar donor, this enzyme adds a β-glucose to ceramide (or N-acylsphingosine; see chemical structure in Figure 1). Once GlcCer is formed, it translocates to the luminal leaflet of Golgi saccules to be further glycosylated and give rise to numerous glycolipids, which are then transported to the plasma membrane.

Enzymatic breakdown of GlcCer in mammalian cells seems to be mediated by at least three β-glucosidases which cleave off the β-glucosidic linkage (see [14]). The best-known GlcCer-degrading enzyme is the acid β-glucosylceramidase (or glucocerebrosidase; GCase), a lysosomal hydrolase encoded by the *GBA1* gene. In the presence of saposin C, the GCase protein catalyzes the degradation of endolysosomal GlcCer, which itself originates from the stepwise degradation of endocytosed glycosphingolipids in the acidic compartments of the cell. The released ceramide then becomes the substrate of the last enzyme of lysosomal sphingolipid catabolism, acid ceramidase (ACDase), which liberates a fatty acid and sphingosine (see Figure 1). In humans and mice, GCase has more recently been shown to catalyze also the transfer of a sterol molecule to β-glucose, thereby forming 1-O-steryl glucoside, as well as some transglucosylation reactions with alcohols [15,16]. While cholesteryl glucoside is a naturally occurring compound, the other transglucosylation products are not.

Gaucher disease (GD) is the most prevalent lysosomal storage disorder involving sphingolipid metabolism; its prevalence is higher in the Ashkenazi Jewish population. It is an autosomal recessive disease, generally caused by pathogenic mutations in the *GBA1* gene (quite exceptionally, it arises from mutations in the *PSAP* gene encoding saposins). By causing the loss of, or a marked reduction in, the catalytic activity of GCase, these mutations are responsible for the lysosomal accumulation of undegraded GlcCer. Importantly, the lysosphingolipid molecule β-glucosylsphingosine (GlcSph) also accumulates [17], likely due to the cleavage of excess GlcCer by lysosomal ACDase [18,19]. The lipid storage mostly affects monocytic-macrophage cells (the so-called Gaucher cells) in the spleen, liver and bone marrow, but can also involve cells of the central nervous system in the most severe, neuronopathic form of the disease. The age of disease onset is extremely variable. The most common subtype of GD is the so-called type 1, with no neurologic involvement. Symptoms of this form of GD include splenomegaly and hepatomegaly, possibly leading to anemia and thrombocytopenia, and bone involvement (osteopenia, fractures, aseptic necrosis and infarcts). Life expectancy in type 1 GD can be normal. Specific treatment of GD is currently based on enzyme replacement therapy, which consists of intravenous infusions of recombinant human GCase every two weeks, or substrate reduction therapy through the oral administration of an inhibitor of GlcCer synthase [20,21,22].

## 2. An increased Risk of Cancer in Patients with Gaucher Disease

In the last thirty years, the association between GD and cancer has been repeatedly described. Indeed, several case studies and small case-series reported on the occurrence of hematologic malignancies in GD, including B-cell or plasma cell malignancy, such as multiple myeloma (MM), acute or chronic leukemia and Hodgkin’s disease [23,24,25,26,27,28]. A causal link between GlcCer storage and occurrence of cancers was already suggested in 1982 by Lee, who found tumors in some of the 239 GD patients examined [29]. In a group of 23 patients, 43% had a diffuse hypergammaglobulinemia and 8% had a monoclonal gammopathy [30]. In a cohort of 63 adult GD patients, a polyclonal gammopathy and monoclonal gammopathy of undetermined significance (MGUS) were observed in 41% and 19% of patients, respectively [31]. MGUS is a pre-malignant condition that predisposes to MM with a 1% risk of transformation per year in the general population [32]. A beneficial effect of enzyme replacement therapy on the development of gammopathies in GD patients has been suggested [31]. Several additional cohorts of GD patients, mostly with type 1 Gaucher, have been studied to further explore the risk of malignancies in such populations; results are summarized in Table 1. Differences between these cohorts include the origin (ethnicity or ancestry) and age of GD patients, and the proportion under enzyme replacement therapy. These possibly explain differences in the overall risk for developing cancers and for the type of malignancies diagnosed. As a matter of fact, the proportion of GD patients with cancer differs from one cohort to another, and the overall relative risk for cancers in GD patients can vary from 0.79 to 3.6. The highest relative risks for a specific type of cancer were reported for hepatocellular carcinoma (up to 141.3), MM (up to 51.1) and hematologic cancers (up to 14.7; [33]) (the reader is also referred to a recent systematic review on this topic [34]). In some studies, such as that published by Zimran and co-workers, 4% of GD patients developed cancers (mainly lymphoma, myelodysplastic syndrome and MM) but there was no statistical difference when comparing with Israeli national registers [35]. Of note, Rosenbloom and co-workers calculated a relative risk of 5.9 for developing MM, but there was no increase in other cancers as compared to the expected incidence in the US population; however, most of the GD patients (85%) studied were under 55 years of age, possibly leading to an underestimation of cancer cases [36]. A very high risk for developing hematologic malignancies, including MM, was also observed in a Western Europe cohort of GD patients [37]. This study also pointed the high prevalence of MGUS (up to 16% in the Dutch GD patients) and underlined the absence of differences in GD activity, spleen status or *GBA1* genotype in patients with or without cancer. Besides hematologic malignancies (non-Hodgkin’s lymphoma, Hodgkin’s disease, acute myeloid leukemia, acute leukemia lymphoid, myelodysplastic syndrome), numerous types of solid tumors have been described in GD patients (see for instance [34,37,38,39]). Interestingly (see Section 4), GD patients display an increased risk of melanoma (relative risk from 2.26 to 3.07) [38,40].

Very recently, Jaffe and co-workers reported that 22% of GD patients from a cohort of 500 Israeli GD patients (quite representative of the Israeli population) had a history of cancer [41]. Patients who received enzyme replacement therapy did not significantly differ from those without this treatment. In this cohort, MM was noted only in five patients (1%).

Among the 658 GD patients of the French GD registry [43], 27 patients with type 1 GD had at least one malignancy: 22 solid tumors and 10 hematologic cancers were observed [42] (Tables S1 and S2). In this group of patients with cancer, 59% received enzyme replacement therapy, again suggesting that the correction of the enzyme deficiency did not reduce the risk of cancer. Moreover, five patients had an additional cancer. The occurrence of multiple cancers in GD has already been reported [38,40,44], some cancers having arisen after splenectomy and/or under enzyme replacement therapy. This observation may strengthen the idea that GD is a predisposing condition for cancer.

## 3. What Could Be the Relationship between Glucosylceramide, Gaucher Disease and Cancer?

GD is commonly associated with chronic cell and tissue inflammation, as well as immune system dysregulation: two factors that could easily be incriminated in the promotion of cancer development. Indeed, analysis of the plasma of patients suffering from GD has revealed increased levels of many pro- and anti-inflammatory cytokines, including IL-1β, TNFα, IL-10 and IL-6 [45,46,47,48], which regulate B-cell proliferation. Interestingly, IL-6 has also been reported to be elevated in GD patients with clonal immunoglobulin abnormalities [45,46]. With regard to the pathogenesis of GD, p38 mitogen-activated protein kinases are believed to act as pro-inflammatory kinases that mediate the production of IL-6 and TNFα [49]. Moreover, administration of the pharmacological chaperone isofagomine to neuronopathic GD mice, which can stimulate mutant GCase function, inhibited p38 activation and TNFα production in brain tissues, and extended animal life span [50]. Which p38 isoforms are predominantly activated in GD patients remains to be determined, although all of them were detected in GD mouse brain [49]. Of particular interest is the observation that ceramide, which is produced by GCase in the PKCδ-dependent salvage pathway [51], down-regulated the activation of the p38δ isoform through ceramide-activated protein phosphatases [52]. Importantly, GBA1 silencing induced p38δ and promoted IL-6 production after phorbol ester treatment [53]. In contrast, increasing the cellular content of ceramide by adding cell-permeable ceramide analogs could reduce IL-6 levels [53]. The altered cytokine profile observed in GD patients may, therefore, be the consequence of abnormal sphingolipid turnover and constitute a favorable environment to promote tumorigenesis.

As far as we know, the pathophysiology of GD is linked to the defective GCase enzymatic activity. Indeed, restoring enzyme activity by exogenous supply of a recombinant active GCase (i.e., enzyme replacement therapy) to the patient corrects most of the non-neurological symptoms (whether the parkinsonism associated with GD or heterozygosity for *GBA1* mutations is solely due to reduced GCase activity or, alternatively, implicates a misfolded GCase protein prone to aggregation, is still a matter of debate). Insufficient GCase activity results in increased levels of undegraded GlcCer and its deacylated form GlcSph in cells, in particular macrophages, known as Gaucher cells, as well as in plasma. Thus, the hypothesis that the glycolipid accumulation in Gaucher cells affects their immune phenotype, leading to alternatively activated (M2) macrophages, was postulated. Quite unexpectedly, the pro-inflammatory cytokine profile does not seem to be the product of Gaucher cells, which instead display anti-inflammatory characteristics with the expression of CD163 and IL1-Ra [45]. However, macrophages surrounding Gaucher cells (for instance, in spleen) would be the source of the pro-inflammatory environment [45]. Additionally, atypical Gaucher cells have been described, which express some markers of tumor-associated macrophages such as CD63, CD168 and VEGF [54], and could participate in tumor development, as already described in hepatocellular carcinoma [55]. Gaucher cells would also produce chemokines leading to an immune cell infiltration (with macrophages, dendritic cells, neutrophils and lymphocytes) in organs, as observed in GD mouse models [56,57,58]. In addition, subcutaneous administration of GlcSph in C57BL/6JRj mice resulted in a splenomegaly characterized by an infiltration of M2 macrophages and a slight elevation in circulating TNFα and IL-1β [59]. Of note, GlcCer has been reported as a specific ligand for macrophage-inducible C-type lectin (Mincle) of antigen-presenting cells, leading to the production of TNFα and MIP-2, as well as the up-regulation of costimulatory molecules presentation [60]. Moreover, the liver of GD mice, treated either by enzyme replacement alone or by substrate reduction therapy (to attenuate sphingolipid storage), showed a decreased macrophage infiltration, suggesting a potential role of GlcCer and GlcSph in controlling the macrophage burden [61].

In GD patients, T-cell-independent activation of B-cells has been reported, which may result from the activation of GlcCer- and GlcSph-specific type II NKT cells constitutively expressing T-follicular helper (T_FH_) phenotype [62]. The clonal immunoglobulins, found in the plasma of GD patients and mice, have been shown to react against GlcSph and GlcCer [63,64]. Interestingly, anti-GlcSph antibodies were reduced in mice treated by eliglustat tartrate (i.e., by substrate reduction therapy) as compared to untreated mice [64]. Moreover, anti-GlcCer IgG autoantibodies and GlcCer could form immune complexes, which activate the complement. While the activation of complement C5a and C5a receptor 1 helped to stimulate pro-inflammatory cytokine production and potentiate the innate and adaptive immune response, it also led to the accumulation of GlcCer by up-regulating the GlcCer synthase [65]. Remarkably, a mouse model of GD with prolonged survival exhibited signs of B-cell hyperproliferation, including lymphadenopathy and plasmacytosis, despite minimal storage of GlcCer [66]. Another strain of mice with an inducible deficiency of GCase in hematopoietic cells, which were characterized by high levels of GlcCer and GlcSph in plasma, liver and spleen, have been reported to develop B-cell lymphoid malignancies. This event was associated with a dysregulation of humoral immunity along with the secretion of clonal immunoglobulins [67]. In this mouse model of non-neuronopathic GD, Gaucher cells were found to infiltrate the splenic T-cell zone and be surrounded by plasma cells. In a similar inducible mouse model of type 1 GD, thymic T development was impaired with a B-cell activation and a recruitment of macrophages and dendritic cells in secondary lymphoid organs [68,69]. The vicinity of Gaucher cells and plasma cells suggests that GlcCer and GlcSph, produced by Gaucher cells, could influence plasma cells. Indeed, when the GD mice were treated early by eliglustat tartrate, leading to a reduced accumulation of these glycolipids, tumor development was prevented; it was only slowed down when treatment was established after tumor development [70]. Hence, through their potential role in regulating chronic antigenic inflammation, immune activation and signaling mechanisms that control cell fate, GlcCer and GlcSph appear to be bona fide candidates for participating in GD predisposition to cancer.

Another hypothetical link between GD and cancer is autophagy, which is critical for cell survival in response to pathologic stresses. GCase has been reported to behave as a positive mediator of autophagic cell death. Indeed, *GBA1* knockdown in A549 lung cancer cells treated with resveratrol, which led to increased intracellular ceramide levels, prevented the abnormal ultrastructural changes characteristic of autophagic cell death [71]. However, defective autophagy has been reported in models of GCase or saposin C deficiency [72,73,74]. Of note, impaired autophagy could be involved in the systemic inflammation discussed above via a constant inflammasome activation in human Gaucher macrophages [75]. An accumulation of GlcCer was also associated to autophagy dysfunction in a drosophila model of GD that lacked the two fly *GBA1* orthologs [76]. In this model, an increase in the autophagolysosomal protein Atg8/LC3 and the ubiquitinated proteins p62 in the fly’s brain was described, leading to age-dependent locomotor deficits and a reduced lifespan. Similarly, autophagy impairment, with an accumulation of autophagosomes and a defect in autophagosome-lysosome fusion, was observed in induced pluripotent stem cells (iPSCs) derived from patients with GD, which exhibit reduced GCase activity and GlcCer accumulation [77].

Interestingly, the inhibition of GCase by conduritol B epoxide was also shown to lead to hyperactivation of the key autophagy inhibitor mTOR and the reduction in levels of the transcription factor EB (TFEB) in a human neuroglioma cell line [78]. TFEB, a master regulator of lysosome function [79], is downregulated by mTORC1 [80] and modulates the expression of genes involved in different steps of the autophagy process [81]. TFEB overexpression alone did not reverse the autophagy defect in iPSCs derived from GD patients, but was effective in the presence of recombinant GCase, suggesting a potential role of GCase on TFEB stability [82]. Altogether, these observations suggest that GCase loss-of-function critically affects functional autophagy. In addition, the pharmacological inhibition of GlcCer synthase led to reduced mTORC1 activation [78], indicating that the lipid GlcCer also regulates autophagy.

While autophagy is often associated with tumor progression, a number of studies have shown that impaired autophagy can favor cancer development [83,84]. First, defective autophagy supports tumorigenesis through mitochondrial dysfunction and the production of mutagenic reactive oxygen species [85,86]. Second, ERK-induced TFEB phosphorylation leads to its inactivation and impairs expression of autophagy-lysosome target genes in BRAF-mutant melanoma [87]. This phenomenon was shown to promote a TGF-β-dependent epithelial-to-mesenchymal transition, metastasis formation and chemoresistance [87]. Third, activation of the mTOR pathway, which participates in protein synthesis and cell proliferation [88], has often been associated with the development of tumors, including melanoma, where it has been correlated with a poor prognosis [89]. Whether disturbed autophagy is causally related to cancer development in GD patients is still unknown, and how GCase affects this process still remains unclear.

A further possible connection between GCase, GD and cancer implicates protein misfolding. It is now established that carriers of *GBA1* mutations have a predisposition to develop Parkinson disease (PD) (see [90,91,92] for reviews). PD is a neurodegenerative disease, associated with the aggregation of misfolded α-synuclein. Intriguingly, PD patients have a higher risk of developing cancer [93], and in particular melanoma [94]. As a matter of fact, melanoma growth was increased in PD mice compared to wild-type mice [95,96]. Several hypotheses have been proposed to explain this finding. A first hypothesis involves the accumulation/aggregation of α-synuclein. Indeed, its expression is higher in the skin of patients with melanoma compared to that of healthy subjects. α-Synuclein could contribute to cancer development by inhibiting autophagy [97]. Its aggregation would result from GCase loss-of-function and, consequently, GlcCer and/or GlcSph accumulation, as shown in PD neuronal cells [98] and in GD/PD mouse models [99]. However, this aggregation may also result from a gain-of-function effect of mutant GCase [90], which could impair the degradation of α-synuclein [100] and other misfolded proteins. These events could be due to disturbances in autophagy and proteasomal degradation [101,102]. A second hypothesis implicates parkin, an ubiquitin E3 ligase involved in the proteasome-dependent degradation of misfolded proteins, and for which loss-of-function mutations underlie familial PD [93]. Parkin is also considered as a tumor suppressor and its loss of function would promote cell proliferation by increasing cyclin E [93]. In GD, endoplasmic reticulum-associated degradation (ERAD) of misfolded mutant GCase has been observed [103]. Mutant GCase could interact directly with parkin in an unnatural manner [104], resulting in the accumulation of its natural substrates [105] and impaired parkin functions. Finally, the accumulation of misfolded proteins in the endoplasmic reticulum activates the unfolded protein response (UPR), which could participate in cancer development [106]. For example, IL-4 synergizes with IL-6 and IL-10 to promote cathepsin secretion in tumor-associated macrophages via UPR activation, resulting in cancer cell invasion [107]. Moreover, in a GD fly model, in which (neuro)inflammation was initially observed, treatment with a GCase chaperone could reverse the phenotype, suggesting a role for GCase in inflammation [108]. Thus, the possibility that mutated GCase by itself could promote cancer by impairing protein degradation systems cannot be ruled out.

## 4. Increased Melanoma Growth in Gaucher Disease

**Figure 2:**
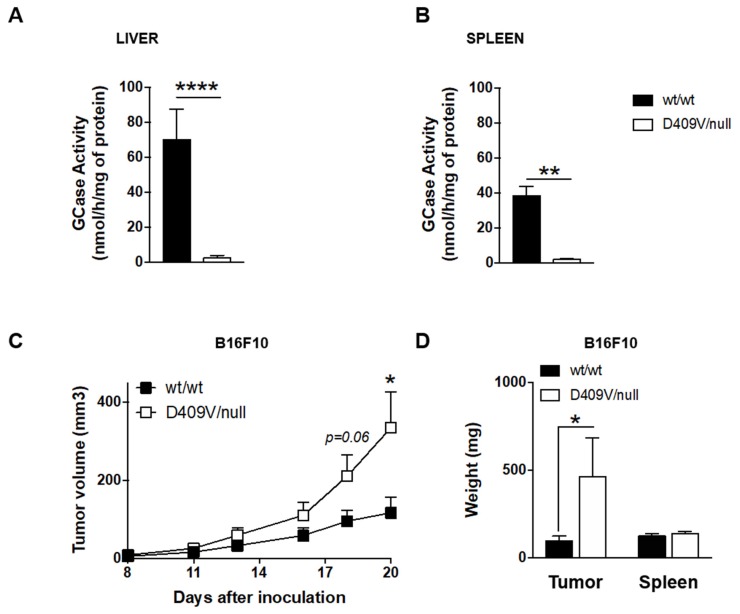
Melanoma tumor growth is increased in Gba1^D409V/null^ mice. (**A**,**B**) GCase enzyme activity was assessed in lysates of liver (**A**) and spleen (**B**) isolated from D409V/null mice (D409V/null) or homozygous wild-type littermates (*wt/wt*) having the same mixed genetic background. Assays were performed in duplicate on samples of three to six animals (18–22 weeks of age). (**C**,**D**) 3 × 10^5^ B16F10 melanoma cells were injected subcutaneously into *wt/wt* or D409V/null mice (19–22 weeks of age). Tumor volumes were measured every 3 days (**C**). Tumor and spleen weights were measured 25 days after tumor inoculation. Data are expressed as means ± SEM of at least two independent experiments (*n* = 7–21 mice). Statistical differences were determined using the Mann–Whitney test. Asterisks indicate statistically significant differences: *, *p* < 0.05; **, *p* < 0.01; ****, *p* < 0.0001.

Based on the above literature, emphasizing the increased risk for GD patients to develop cancer, we wished to experimentally assess such a risk in an animal model recapitulating the main features of type 1 GD. In this experimental approach, a skin melanoma model was studied, as the prevalence of this type of cancer has been shown to be more frequent in GD patients (see Table 1). To this end, compound heterozygous *Gba1^D409V/null^* mice and age-matched wild-type littermates were challenged with a subcutaneous injection of the B16F10 mouse melanoma cell line (i.e., a cell line derived from animals with the C57BL/6 background). These compound heterozygous GD mice, which were previously obtained by crossing *Gba1^D409V/D409V^* mice with *Gba1^wt/null^* mice, exhibited 3% ± 2% and 5% ± 1% residual GCase activity in liver (Figure 2A) and spleen (Figure 2B), respectively, compared to wild-type mice. These mice have been shown to accumulate GlcCer in liver and spleen [56,109], pointing to the association of the observed deficient GCase activity, and the storage of GlcCer as well as GlcSph, as reported in patients with GD [110,111].

Analysis of tumor volume (Figure 2C) and tumor weight (Figure 2D) showed that the B16F10 cells grew much faster in *Gba^D409V/null^* mice than in wild-type animals bearing the same tumors. In contrast, spleen weight remained unchanged (Figure 2D). Similar observations were made using a distinct GD mouse model, the homozygous *Gba^D409V/D409V^*, known to accumulate both GlcCer and GlcSph [112], and using a distinct melanoma cell line, B16BL6 [113].

Altogether, these data suggest that the sphingolipid storage in host tissues, which is associated with homozygosity or compound heterozygosity for disease-causing mutations in the *Gba1* gene, could favor melanoma tumor growth in mice. While no significant simple changes in the tumor-infiltrating lymphoid cells have yet been detected in our GD/melanoma model [113], further investigations are necessary, especially in order to carefully characterize the serum cytokines and chemokines as well as the type of immune cells in blood and in tumors. As previously alluded to, the GD mice we used are known to exhibit systemic inflammation with a complex pattern of infiltration of immune cells in organs, including both IFNγ-regulated pro-inflammatory and IL-4-regulated anti-inflammatory networks, which can be reversed by enzyme replacement therapy [57,58,61,65,114]. In addition, the systemic elevation of some cytokines, such as TNFα, IL-6, IL-8, and IL-10, observed in GD patients [46,48,115], which are known to promote tumor development, could be involved in the increased tumor growth we observed.

## 5. Concluding Remarks and Outlook

Accumulating evidence, based on both clinical observations on large patient populations and animal and in vitro studies, highlights the link between GD and cancer. The involvement of undegraded substrates, i.e., GlcCer and GlcSph, and the subsequent alterations in the inflammatory and immune responses seem to be the most legitimate underlying hypotheses. The resulting changes in the tumor environment may well contribute to tumor development. However, how the GCase lipid substrates finely modulate signaling pathways and promote cancer development still remains unclear. It has been postulated that, following its formation by ACDase, GlcSph could exit the lysosomal compartment and be degraded by the non-lysosomal β-glucosidase GBA2, thereby releasing sphingosine, which could then be phosphorylated to produce sphingosine 1-phosphate (see Figure 1). The latter lysolipid is well known for its oncogenic properties, such as tumor progression, inflammation, and immune cell trafficking [3,116]. Supporting this tenet, Mistry and co-workers generated a deletion of *Gba2* in their GD mouse model, which led to some rescue of the phenotype, suggesting a role for these sphingolipid products. However, the Th1 and Th2 cytokine profiles were not completely restored, indicating that GlcCer and GlcSph, which remained elevated, play an intrinsic role [117]. Additional GlcCer- and GlcSph-derived sphingolipids could be implicated in the increased risk for lymphoproliferative disorders and cancers that are associated with GD. For instance, GM3 levels are increased in GD [17]. This ganglioside, present in many tumor types, is generally viewed as an inhibitory or suppressive agent in cancer development and progression [4]. Whether it interferes with cancer cell development in GD is entirely unknown.

An important question arises as to whether current strategies used to treat GD can influence the development and onset of associated malignancies. At this stage, GD patients under enzyme replacement therapy analyzed in several cohorts (see Table 1 and Tables S1 and S2) do not seem to have a lower risk of developing cancer. It is still difficult to exclude the possibility that therapies used for GD may have not been long enough to fully correct all the abnormalities leading to cancer. In addition, which GD biomarker is associated with any possible accompanying cancer is unknown. Future studies on GD patients receiving disease-specific treatment (either enzyme replacement therapy or substrate reduction therapy) should carefully monitor relevant biological markers such as plasma sphingolipids, i.e., GlcCer, GlcSph, and ideally sphingosine and sphingosine 1-phosphate, as well as cytokines/chemokines, to assess any potential correlation between these biomarkers and the occurrence of malignancies. Elucidation of the potential roles of GlcCer-related sphingolipids in driving the increased risk of cancer in GD patients is of utmost importance to define novel therapeutic strategies. For instance, should GlcSph be causally involved, as postulated for GD-associated gammopathies and multiple myeloma [64], ACDase inhibitors may prove to be valuable. In a similar way, should the non-lysosomal GBA2 and sphingosine 1-phosphate be implicated, enzyme inhibitors of GBA2 and/or sphingosine kinases may be attractive strategies for the prevention of cancers in GD patients. The relevance of such combinations is supported by the observation that the deletion of Gba2 ameliorates the phenotype of GD type 1 mice [117]. The assessment and possible validation of these hypotheses undoubtedly needs the development of appropriate animal models.

## Figures and Tables

**Table 1:** Susceptibility of patients affected with Gaucher disease to develop malignancies.

Type of Population	Number of Patients	Age of Patients (Years)	Incidence and Type of Cancer, and Relative Risk for the Indicated Malignancy (in Brackets, 95% CI)	Overall Relative Risk for Cancer (in Brackets, 95% CI)	Reference
Ashkenazi Jewish (Israel)	48	54 ± 20	Patients with any cancer: 20.8% Mean age at cancer diagnosis: 57 ± 18 RR for hematologic cancers: 14.7 [5.2–41.7]	3.6 [1.7–7.5]	Shiran et al. 1993 [33]
Caucasian, non-Jewish (22.7%) Ashkenazi Jewish (23.2%) Unreported (39.8%)	2742	0–14: 25.6% 15–44: 47% 45–54: 12.2% 55–64: 7.5% >65: 7.7%	Patients with any cancer: 4.6% (9.1% in Ashkenazi Jewish) RR for myeloma: 5.9 [2.8–10.8] RR for hematologic cancers: 1.23 [0.73–1.90]	0.79 [0.67–0.94]	Rosenbloom et al. 2005 [36]
Ashkenazi Jewish	505	Males: 38.7 ± 21 Females: 37 ± 21	Patients with any cancer: 4.0%	Males: 0.6 [0.12–1.1] Females: 1.4 [0.7–2.2]	Zimran et al. 2005 [35]
Mostly non-Ashkenazi Jewish (Netherlands and Germany)	131	50 ± 14	Patients with any cancer: 10.7% Median age at cancer diagnosis: 52 RR for hematologic cancers: 12.7 [2.6–37.0] RR for myeloma: 51.1 [6.2–184] RR for liver carcinoma: 141.3 [17.1–510.5]	2.5 [1.1–4.7]	de Fost et al. 2006 [37]
US male veterans (African Americans: 11.7%)	1525	African Americans: 46.0 Whites: 49.7	Patients with any cancer: 9% RR for non-Hodgkin lymphoma: 2.54 [1.32–4.88] RR for melanoma: 3.07 [1.28–7.38] RR for pancreatic cancer: 2.37 [1.13–4.98]	0.91 [0.76–1.08]	Landgren et al. 2007 [40]
Mostly Ashkenazi Jewish	403	44 (for homozygous N370S patients)	Patients with any cancer: 12.5% RR for myeloma: 25 [9.17–54.4] RR for other hematologic cancers: 3.45 [1.49–6.79] RR for breast cancer: 1.84 [0.84–3.49] RR for melanoma: 2.26 [0.62–5.79]	1.80 [1.32–2.40]	Taddei et al. 2009 [38]
Mostly Jewish (Israel)	500	0–17: 9.4% 18–24: 4.4% 25–34: 11.4% 35–44: 20.6% 45–54: 12.4% 55–64: 16.0% 65–74: 14.6% >75: 11.2%	Patients with any cancer: 22.2% Patients with myeloma: 1%	ND	Jaffe et al. 2019 [41]
Caucasian (France)	658	50.8	Patients with any cancer: 4.1% Patients with hematologic cancers: 1.5%	ND	Stirnemann (unpublished) [42]

Abbreviations: CI, confidence intervals; ND, not described; RR, relative risk.

## Supplementary Materials

The following are available online at https://www.mdpi.com/2072-6694/12/2/475/s1, Table S1: Characteristics of cancer patients in the French Registry of Gaucher Disease (total: 658 GD patients), Table S2: Characteristics of cancers in the French Registry of Gaucher disease.

## Abbreviations

ACDase: acid ceramidase; GCase, glucosylceramidase; GD, Gaucher disease; GlcCer, β-glucosylceramide; GlcSph, β-glucosylsphingosine; iPSCs, induced pluripotent stem cells; MGUS, monoclonal gammopathy of undetermined significance; MM, multiple myeloma; PD, Parkinson disease; TFEB, transcription factor EB; UPR, unfolded protein response.
