# Supplementary material for: Are Glucosylceramide-Related Sphingolipids Involved in the Increased Risk for Cancer in Gaucher Disease Patients? Review and Hypotheses
Source: Cancers (Basel). 2020 Feb 18;12(2):475. doi: 10.3390/cancers12020475 (PMC7072201; doi:10.3390/cancers12020475)
Supplement: Supplementary file 1 [file cancers-12-00475-s001.pdf]

Table S1: Characteristics of cancer patients in the French Registry of Gaucher Disease (total: 658 GD patients).

		Number of patients studied
GD patients included		27
Median age of first symptoms (years; min-max)	35 (6 – 64)	14
Median time of follow-up of GD (years; min-max)	31.3 (5.3 – 74.3)	27
Sex ratio	0.42	27
Patients with additional case among relatives	9	27
Gaucher type 1	27	27
Patients without genotype	7	27
Patients with full genotype	17	27
N370S/N370S	2	20
L444P/L444P	0	20
L444P/N370S	4	20
N370S/other	9	20
L444P/other	0	20
other/other	5	20
Patients with splenectomy	12	27
Patients with >1 bone event	18	26
Patients previously treated prior to inclusion	16	27
Treatment duration (years)	1 (0.1-7.3)	16
Imiglucerase	12	16
Velaglucerase	1	16
Alglucerase	2	16
Miglustat	1	16

Table S2: Characteristics of cancers in the French Registry of Gaucher disease.

Patient	First cancer	Age at first cancer (years)	Second cancer	Age at second cancer (years)	Age at diagnosis of GD (years)	Age at splenectomy (years)	First treatment of GD	Age at first treatment (years)	Age of death (years)
1	Basal cell carcinoma (skin)	67.3			6.3	6.3	Alglucerase	53.3	73.9
2	Cholangiocarcinoma	72.0			9.0	18.0	Alglucerase	48.5	
3	Smoldering myeloma	67.7			33.7	NA	Imiglucerase	51.7	
4	Endometrial carcinoma	62.6	Angioimmunoblastic T cell lymhoma	83.0	61.6	NA	Miglustat	79.0	83.4
5	Retractory anemia (myelodysplastic disease)	49.4			28.7	29.7	Imiglucerase	50.3	68.9
6	Thyroid cancer	34.2	Lymphoma	56.8	0.0	31.2	Imiglucerase	45.7	56.8
7	Lung adenocarcinoma	62.3			45.4	NA	Imiglucerase	50.7	
8	intraductal breast cancer	NA			63.6	NA	none		
9	Ductal breast cancer	42.0			33.5	NA	Imiglucerase	43.4	
10	Basal cell carcinoma (skin)	NA			53.7	NA	Imiglucerase	54.2	
11	Malt lymphoma	47.9	Thyroid papillar carcinoma	62.3	24.3	24.3	Imiglucerase	51.3	
12	Chronic lymphocytic leukemia	61.2	Endometrial carcinoma	62.2	56.9	61.7	none		67.1
13	Thyroid cancer	31.7			25.7	NA	none		
14	Essential thrombocytemia JAK2-mutated	65.9			11.9	15.9	Imiglucerase	59.5	
15	Lung cancer	57.2			42.4	NA	none		57.7
16	Squamous cell carinoma (skin)	80.3	Lung adenocarcinoma	85.7	59.8	NA	none		
17	Malt Lymphoma	65.8			34.1	NA	Velaglucerase	64.6	
18	Bladder cancer	70.9			29.9	29.9	Imiglucerase	70.9	
19	Colonic cancer				44.4	NA	none		
20	Prostate cancer	65.0			57.0	NA	Imiglucerase	74.0	76.9
21	Osteocarcoma	58.2			34.2	34.2	none		69.2
22	Multiple myeloma	71.5			62.4	no	none		
23	Lung squamous cell carcinoma	67.8			36.2	36.2	Imiglucerase	64.7	68.2
24	Metastatic ovarian cancer	72.2			67.4	67.6	Imiglucerase	67.5	

25	Adenocarcinoma	31.9			53.1	53.1	none		
26	Myeloma	84.8			46.0	no	none		
27	Skin carcinoma	72.1			72.1	no	none		

NA, not available.
